# Clinical Phenotype and Prognosis of Asymptomatic Patients With Transthyretin Cardiac Amyloid Infiltration

**DOI:** 10.1001/jamacardio.2024.5221

**Published:** 2025-01-22

**Authors:** Aldostefano Porcari, Yousuf Razvi, Francesco Cappelli, Christian Nitsche, Matteo Serenelli, Simone Longhi, Giulio Sinigiani, Alberto Cipriani, Alberto Aimo, Daniela Tomasoni, Mattia Zampieri, Anna Cantone, Valentina Allegro, Giuseppe Vergaro, Ahmad Masri, Marcus Urey, Adam Ioannou, Aviva Petrie, Navid Noory, Finn Gustafsson, Michael Poledniczek, Michele Emdin, Marco Metra, Gianfranco Sinagra, Ana Martinez-Naharro, Ashutosh D. Wechalekar, Helen Lachman, Carol Whelan, Philip N. Hawkins, Scott D. Solomon, Julian D. Gillmore, Marianna Fontana

**Affiliations:** 1 National Amyloidosis Centre, Division of Medicine, University College London, Royal Free Hospital, London, United Kingdom; 2 Center for Diagnosis and Treatment of Cardiomyopathies, Cardiovascular Department, Azienda Sanitaria Universitaria Giuliano-Isontina, University of Trieste, Trieste, Italy; 3 European Reference Network for Rare, Low Prevalence and Complex Diseases of the Heart, ERN GUARD-Heart, Trieste, Italy; 4 Cardiomyopathy Unit, Careggi University Hospital, University of Florence, Florence, Italy; 5 Tuscan Regional Amyloidosis Centre, Careggi University Hospital, Florence, Italy; 6 Division of Cardiology, Department of Internal Medicine II, Medical University of Vienna, Vienna, Austria; 7 Cardiologic Centre, University of Ferrara, Cona, Italy; 8 Cardiology Unit, Istituto di Ricovero e Cura a Carattere Scientifico Azienda Ospedaliero-Universitaria di Bologna, Bologna, Italy; 9 Department of Cardiac, Thoracic and Vascular Sciences and Public Health, University of Padua, Padua, Italy; 10 Institute of Life Sciences, Scuola Superiore Sant’Anna, Pisa, Italy; 11 Cardiology Division, Fondazione Toscana Gabriele Monasterio, Pisa, Italy; 12 Cardiology, Azienda Socio Sanitaria Territoiale Spedali Civili, Department of Medical and Surgical Specialties, Radiological Sciences and Public Health, University of Brescia, Brescia, Italy; 13 Knight Cardiovascular Institute, Oregon Health & Science University, Portland, Oregon; 14 Division of Cardiovascular Diseases, Department of Medicine, University of California, San Diego, La Jolla; 15 Department of Cardiology and Clinical Medicine, Rigshospitalet, University of Copenhagen, Copenhagen, Denmark; 16 Cardiovascular Division, Department of Medicine, Brigham and Women’s Hospital, Boston, Massachusetts

## Abstract

**Importance:**

Patients with transthyretin (ATTR) cardiac amyloid infiltration are increasingly diagnosed at earlier disease stages with no heart failure (HF) symptoms and a wide range of cardiac amyloid infiltration.

**Objective:**

To characterize the clinical phenotype and natural history of asymptomatic patients with ATTR cardiac amyloid infiltration.

**Design, Setting, and Participants:**

This cohort study analyzed data of all patients at 12 international centers for amyloidosis from January 1, 2008, through December 31, 2023. Inclusion criteria were asymptomatic ATTR cardiac amyloid infiltration, defined as an absence of HF history, HF signs and symptoms, diuretic therapy, and plasma cell dyscrasia with evidence of myocardial uptake on bone scintigraphy. If plasma cell dyscrasia was present, histologic confirmation of ATTR amyloid was required.

**Exposure:**

Asymptomatic ATTR cardiac amyloid infiltration.

**Main Outcomes and Measures:**

The primary outcomes were all-cause and cardiovascular (CV) mortality. The secondary outcomes were unplanned HF hospitalization, unplanned CV-related hospitalization, and a composite outcome of CV mortality and HF hospitalization.

**Results:**

The study comprised 485 patients with asymptomatic ATTR cardiac amyloid infiltration (mean [SD] age, 74.9 [9.9] years, 85.8% male, 112 [23.1%] with hereditary ATTR amyloidosis), with 369 (76.1%) having grade 2 or 3 and 116 (23.9%) having grade 1 cardiac uptake at baseline. Patients with grade 2 or 3 uptake exhibited significantly more cardiac functional and structural abnormalities vs patients with grade 1 uptake. At 3 years, compared with grade 1 uptake, patients with grade 2 or 3 uptake had greater development of HF (54.3% [95% CI, 47.7%-61.3%] vs 23.1% [95% CI, 14.8%-35.1%]), greater outpatient diuretic initiation and N-terminal pro-B-type natriuretic peptide progression (35.0% [95% CI, 28.0%-43.2%] vs 12.4% [95% CI, 6.3%-23.7%]), and greater HF hospitalization (8.7% [95% CI, 5.9%-12.9%] vs 0%) and unplanned CV hospitalization (20.0% [95% CI, 15.7%-25.3%] vs 4.3% [95% CI, 1.6%-11.3%]). Over a median follow-up of 37 months (IQR, 20-64 months), the all-cause death rate was similar between patients with grade 1 vs 2 and 3 uptake; however, those with grade 2 or 3 compared with grade 1 uptake had a significantly higher risk of CV mortality (unadjusted hazard ratio, 5.30; 95% CI, 1.92-14.65).

**Conclusions and Relevance:**

This study shows that asymptomatic ATTR cardiac amyloid infiltration encompasses a wide spectrum of disease severity, with patients with grade 2 or 3 cardiac uptake experiencing an increased rate of CV events and CV mortality and patients with grade 1 uptake experiencing a lower CV event rate and predominantly non-CV mortality. These findings support the use of disease-modifying treatments in asymptomatic patients with grade 2 or 3 uptake and highlight the need of large-scale studies to assess their role in grade 1 uptake.

## Key Points

**Question:**

What is the clinical phenotype and natural history of patients with asymptomatic transthyretin (ATTR) cardiac amyloid infiltration?

**Findings:**

In this cohort study of 485 patients with asymptomatic ATTR cardiac amyloid infiltration, those with grade 2 or 3 cardiac uptake had greater rates of disease progression and a 5-fold higher risk of cardiovascular death compared with those with grade 1 uptake, in whom noncardiovascular death was predominant.

**Meaning:**

These findings support the use of disease-modifying treatments in patients with ATTR cardiac amyloid infiltration with grade 2 and 3 uptake and highlight the need for large-scale studies to assess their role in grade 1 uptake.

## Teaser

This cohort study characterizes the clinical phenotype and natural history of asymptomatic patients with transthyretin cardiac amyloid infiltration.

## Introduction

Transthyretin amyloid cardiomyopathy (ATTR-CM) is caused by the progressive accumulation of insoluble transthyretin amyloid fibrils in the myocardial extracellular space.^1^ It is thought to result from age-related failure of homeostatic mechanisms in wild-type ATTR-CM (nonhereditary form) or destabilizing mutations in variant ATTR-CM (hereditary form).^1^

ATTR-CM is now recognized as a relatively prevalent cause of heart failure (HF) worldwide. Major advances in noninvasive imaging techniques in recent years have led to a greater number of patients being identified earlier in the disease process, with an increasing proportion of the patients exhibiting evidence of asymptomatic ATTR cardiac amyloid infiltration.^2^ However, there is a paucity of data on the phenotypic characterization and natural history of asymptomatic ATTR cardiac amyloid infiltration, particularly concerning disease progression and mortality.^3,4^ Only 1 study, with 118 patients, has reported outcome data in asymptomatic patients, but it included a heterogenous population with a high proportion of patients lacking bone scintigraphy and nearly one-half receiving disease-modifying treatments.^3^ Tafamidis (ATTR-ACT trial),^5^ acoramidis (ATTRIBUTE-CM trial),^6^ and vutrisiran (HELIOS-B trial)^7^ have demonstrated benefits in patients with ATTR-CM and HF symptoms, but patients with asymptomatic cardiac amyloid infiltration have been excluded from all clinical trials of disease-modifying treatment. Whether earlier intervention could result in better outcomes in ATTR-CM remains unknown,^8^ as does the optimal point in the patient’s disease progression for the use of disease-modifying treatments. Understanding the natural history of asymptomatic cardiac amyloid infiltration is essential for making informed clinical decisions at the patient level and for designing randomized clinical trials in this population. The aim of our study was to characterize the clinical phenotype and natural history of patients with asymptomatic ATTR cardiac amyloid infiltration.

## Methods

### Patient Population

We conducted a multicenter, international retrospective cohort study of patients diagnosed with ATTR cardiac amyloid infiltration between January 1, 2008, and December 31, 2023, from 12 specialist referral centers: National Amyloidosis Centre, London, UK; Portland, Oregon; San Diego, California; Vienna, Austria; Copenhagen, Denmark; and 7 Italian centers (Trieste, Bologna, Ferrara, Florence, Brescia, Padua, and Pisa). Local institutional review board approval for the study was obtained from each participating center. The study was conducted in adherence to the Declaration of Helsinki,^9^ and written informed consent for publication of the patients’ anonymized data was obtained under the institutional review board policies from the relevant individual specialist centers. The study followed the Strengthening the Reporting of Observational Studies in Epidemiology (STROBE) reporting guideline.

Diagnosis of ATTR cardiac amyloid infiltration was established on the basis of myocardial uptake on bone scintigraphy (ie, grade 1, 2, or 3, confirmed by single-photon emission tomography/computed tomography imaging) (1) in the absence of biochemical evidence of a plasma cell dyscrasia (PCD) or (2) biopsy proof of ATTR amyloid in the presence of PCD.^10^ Patients with asymptomatic ATTR cardiac amyloid infiltration, defined as the absence of HF history, HF signs and symptoms, and diuretic therapy at diagnosis, were eligible for inclusion in the analysis. All patients underwent sequencing of the *TTR* gene.^11^ All echocardiographic parameters were measured according to standard international definitions.^12,13^ All patients were enrolled into a protocolized clinical follow-up program at participating centers with clinical assessment, laboratory tests, and echocardiography.^14^

### Definition of Symptomatic HF and HF-Related Criteria of Disease Progression

Development of symptomatic HF in the outpatient setting was defined as progression to a New York Heart Association functional class of 2 or higher that would require initiation of diuretic therapy.^3^ Outpatient diuretic initiation (ODI) was defined as new initiation of a loop diuretic agent in diuretic-naive patients.^15^ N-terminal pro-B-type natriuretic peptide (NT-proBNP) progression was defined as an absolute increase of more than 700 ng/L and a relative percent increase of more than 30% compared with baseline.^15^

### Outcomes

The primary outcomes of the study were all-cause and cardiovascular (CV) mortality. Secondary outcome measures were (1) unplanned hospitalization for worsening HF, (2) unplanned CV-related hospitalization, and (3) a composite outcome of CV mortality or HF hospitalization. The mortality end point was defined as time to death from baseline for all deceased patients and time to censor date (June 30, 2024) from baseline among the remainder. Cardiovascular mortality was defined as sudden cardiac death, aborted cardiac death, fatal stroke, fatal cardiac transplant, or end-stage HF with pump failure. Data on HF hospitalizations and ODI were obtained from scheduled follow-up evaluations at participating centers and from electronic health record systems.

### Statistical Analysis

Descriptive statistic analyses between the study groups were performed. All continuous variables were tested for normality (Shapiro-Wilk test) and are presented as mean with standard deviation if the distribution was normal or median with interquartile range otherwise. Categorical variables are expressed as absolute numbers and frequencies. The 2-sample *t* test for continuous variables was used to compare means if the data were normally distributed in each treatment group, and its nonparametric equivalent, the Mann-Whitney *U* test, was used otherwise to compare the distributions of the 2 treatment groups. The χ^2^ test or Fisher exact test was used for categorical variables. Survival was evaluated using Cox proportional hazards regression analysis, providing estimated hazard ratios (HRs) with 95% CIs and Kaplan-Meier curves. The proportional hazards assumption was checked and confirmed.

All statistical analyses were performed using SPSS, version 26.0 (IBM Corporation) and Stata, version 15 (StataCorp LLC). A 2-sided *P* < .05 was considered statistically significant.

## Results

A total of 660 asymptomatic patients were identified (eTable 1 in Supplement 1). During follow-up, 175 (26.5%) patients were enrolled into interventional ATTR-CM clinical trials (52 patients [29.7%]) or received disease-modifying therapy (tafamidis, 102 patients [58.3%]; patisiran, 21 patients [12.0%]) and were excluded from this analysis.

The study population comprised 485 asymptomatic patients (mean [SD] age, 74.9 [9.9] years; 69 female [14.2%] and 416 male [85.8%]) of whom 373 (76.9%) had wild-type and 112 (23.1%) had variant-type ATTR amyloidosis (Table 1). A total of 116 patients (23.9%) had grade 1 myocardial uptake (31 [26.7%] with histology demonstrating ATTR amyloid, 87 [75.0%] without PCD), and 369 patients (76.1%) had grade 2 or 3 myocardial uptake (67 [18.2%] with histology demonstrating ATTR amyloid, 302 [81.8%] without PCD). The number of asymptomatic patients with ATTR cardiac amyloid infiltration at diagnosis was 26 (5.4%) in 2008-2011, 60 (12.4%) in 2012-2015, 157 (32.4%) in 2016-2019, and 242 (49.9%) in 2020-2023. Patients with grade 1 uptake were older (eFigure 3 in Supplement 1), showed less abnormal cardiac structure and function, and had normal or only mildly elevated NT-proBNP concentrations (median, 278 ng/L; IQR, 94-923 ng/L). However, patients with grade 2 or 3 uptake had structural and functional features on echocardiogram typical of amyloid infiltration, which were associated with significantly elevated serum NT-proBNP concentrations (median, 650 ng/L; IQR, 320-1095 ng/L; *P* < .001) and a higher rate of atrial fibrillation (30.4% [112 patients] vs 19.8% [23 patients with grade 1 uptake]; *P* = .03).

**Table 1.** Baseline Characteristics of the Study Population

Parameter	No. missing	No. (%)			*P* value
		All (N = 485)	Perugini grade 1 (n = 116)	Perugini grade 2-3 (n = 369)	
Age, mean (SD), y	0	74.9 (9.9)	74.0 (12.0)	75.2 (9.0)	.91
Sex					
Female	0	69 (14.2)	24 (20.7)	45 (12.2)	.02
Male	0	416 (85.8)	92 (79.3)	324 (87.8)	
SBP, median (IQR), mm Hg	10	132 (121 to 147)	132 (122 to 149)	131 (120 to 146)	.38
Wild-type ATTR	0	373 (76.9)	80 (69.0)	293 (79.4)	.02
Hereditary ATTR	0	112 (23.1)	36 (31.0)	76 (20.6)	.02
Atrial fibrillation	0	135 (27.8)	23 (19.8)	112 (30.4)	.03
IHD	0	82 (16.9)	19 (16.4)	63 (17.1)	.86
Diabetes	0	60 (12.4)	16 (13.8)	44 (11.9)	.59
Hypertension	0	223 (46.0)	43 (37.1)	180 (48.8)	.03
Previous stroke or TIA	0	34 (7.0)	6 (5.2)	28 (7.6)	.37
Heart failure severity					
NYHA class					
1	0	485 (100)	116 (100)	369 (100)	NA
2	0	0 (0)	0 (0)	0 (0)	
3	0	0 (0)	0 (0)	0 (0)	
4	0	0 (0)	0 (0)	0 (0)	
NAC stage					
1a	0	264 (54.4)	76 (65.5)	188 (50.9)	.01
1b	0	194 (40.0)	35 (30.2)	159 (43.1)	
2	0	26 (5.4)	4 (3.4)	22 (6.0)	
3	0	1 (0.2)	1 (0.9)	0 (0)	
NT-proBNP, median (IQR), ng/L	0	587 (265 to 1062)	278 (94 to 923)	650 (320 to 1095)	<.001
eGFR, median (IQR), mL/min/1.73 m^2^	0	74 (59 to 86)	74 (62 to 89)	74 (58 to 85)	.26
Echocardiographic parameters					
IVS, mean (SD), mm	0	15.1 (3.3)	12.6 (2.8)	16.0 (3.0)	<.001
PW, mean (SD), mm	0	13.8 (2.9)	11.6 (2.6)	14.5 (2.7)	<.001
RWT, median (range)	90	0.66 (0.50 to 0.82)	0.47 (0.41 to 0.60)	0.70 (0.56 to 0.83)	<.001
LVEF, mean (SD), %	0	57.5 (8.7)	58.9 (8.9)	57.1 (8.7)	.02
LVEF <50%	0	77 (15.9)	11 (9.5)	66 (17.9)	.03
LVEF ≤40%	0	22 (4.5)	4 (3.4)	18 (4.9)	.51
LV-GLS, median (IQR), %	60	−15.9 (−12.5 to −19.0)	−19.0 (−16.0 to −20.2)	−14.9 (−12.0 to −18.0)	<.001
E/e′, mean (SD)	49	12.3 (4.7)	9.7 (4.0)	13.2 (12.6)	<.001
LA area, mean (SD), cm^2^	63	24.3 (8.9)	21.3 (7.0)	25.3 (9.3)	<.001
RA area, mean (SD), cm^2^	74	20.4 (7.0)	17.1 (4.5)	19.9 (6.0)	.001
TAPSE, mean (SD), mm	55	20.4 (3.8)	21.1 (2.8)	20 (4.1)	.003
Medications					
Loop diuretic	0	0	0	0	NA
ACEi, ARB, ARNI	0	180 (37.1)	23 (19.8)	157 (42.5)	<.001
β-Blocker	0	127 (26.2)	18 (15.5)	109 (29.5)	.003
MRA	0	18 (3.7)	0	18 (4.9)	.02
SGLT2i	0	11 (2.3)	3 (2.6)	8 (2.2)	.79

Abbreviations: ACEi, angiotensin-converting enzyme inhibitor; ARB, angiotensin receptor blocker; ARNI, angiotensin receptor-neprilysin inhibitor; ATTR, transthyretin; E/e′, ratio of mitral inflow velocity to mitral annular velocity; eGFR, estimated glomerular filtration rate; IHD, ischemic heart disease; IVS, interventricular septal thickness; LA, left atrium; LVEF, left ventricular ejection fraction; LV-GLS, left ventricular global longitudinal strain; MRA, mineralocorticoid receptor agonist; NA, not applicable; NAC, National Amyloidosis Center; NT-proBNP, N-terminal pro-B-type natriuretic peptide; NYHA, New York Heart Association; PW, posterior wall thickness; RA, right atrium; RWT, relative wall thickness; SBP, systolic blood pressure; SGLT2i, sodium-glucose cotransporter 2 inhibitor; TAPSE, tricuspid annular plane systolic excursion; TIA, transient ischemic attack.

Asymptomatic individuals were referred for cardiac scintigraphy due to suggestive cardiac imaging features identified during routine monitoring of their cardiologic comorbidities (266 patients [54.8%]; 24 patients with grade 1 [20.7%]; 242 [65.6%] patients with grade 2 or 3), incidental cardiac uptake on scintigraphy or extracardiac histologic proof of ATTR amyloid performed for noncardiac reasons (153 patients [31.5%]; 61 with grade 1 [52.6%]; 92 with grade 2 or 3 [24.9%]), detection of a pathogenic TTR variant through *TTR* gene sequencing prompted by a family history of hereditary amyloid (51 patients [10.5%]; 25 with grade 1 [21.6%]; 26 with grade 2 or 3 [7.0%]), and suggestive neurologic symptoms accompanied by either histologic evidence of ATTR amyloid or identification of a pathogenic TTR variant (15 patients [3.1%]; 6 with grade 1 [5.2%]; 9 with grade 2 or 3 [2.4%]). In particular, among patients with grade 1 cardiac uptake, 16 of 61 (26.2%) underwent bone scintigraphy or extracardiac biopsy as part of diagnostic workup for cancer.

### Survival Outcomes Among Asymptomatic Patients With ATTR Cardiac Amyloid Infiltration

**Figure 1.**
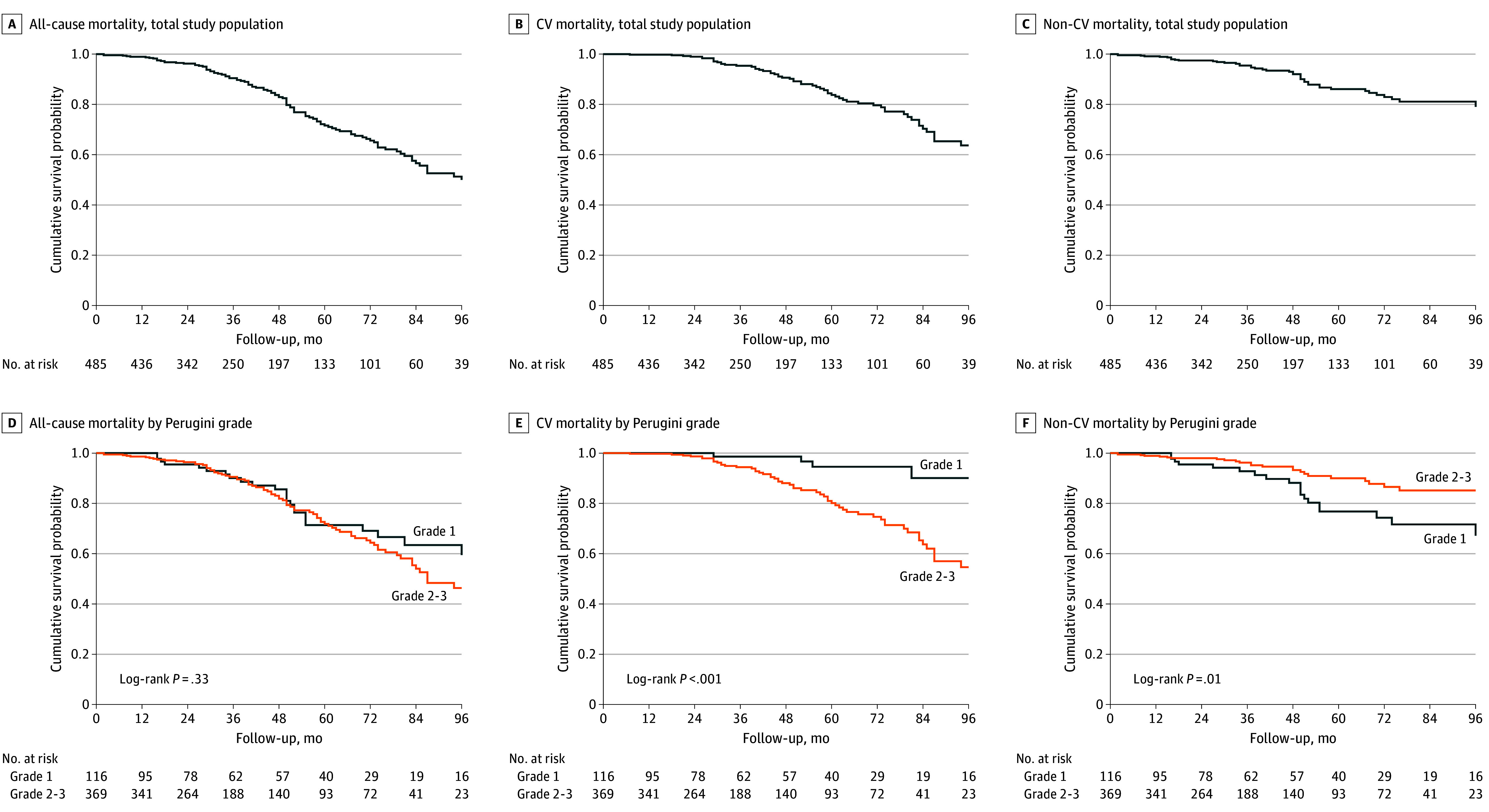
Mortality for the Total Study Population and According to Perugini Grade of Cardiac Uptake at Diagnosis CV indicates cardiovascular.

**Figure 2.**
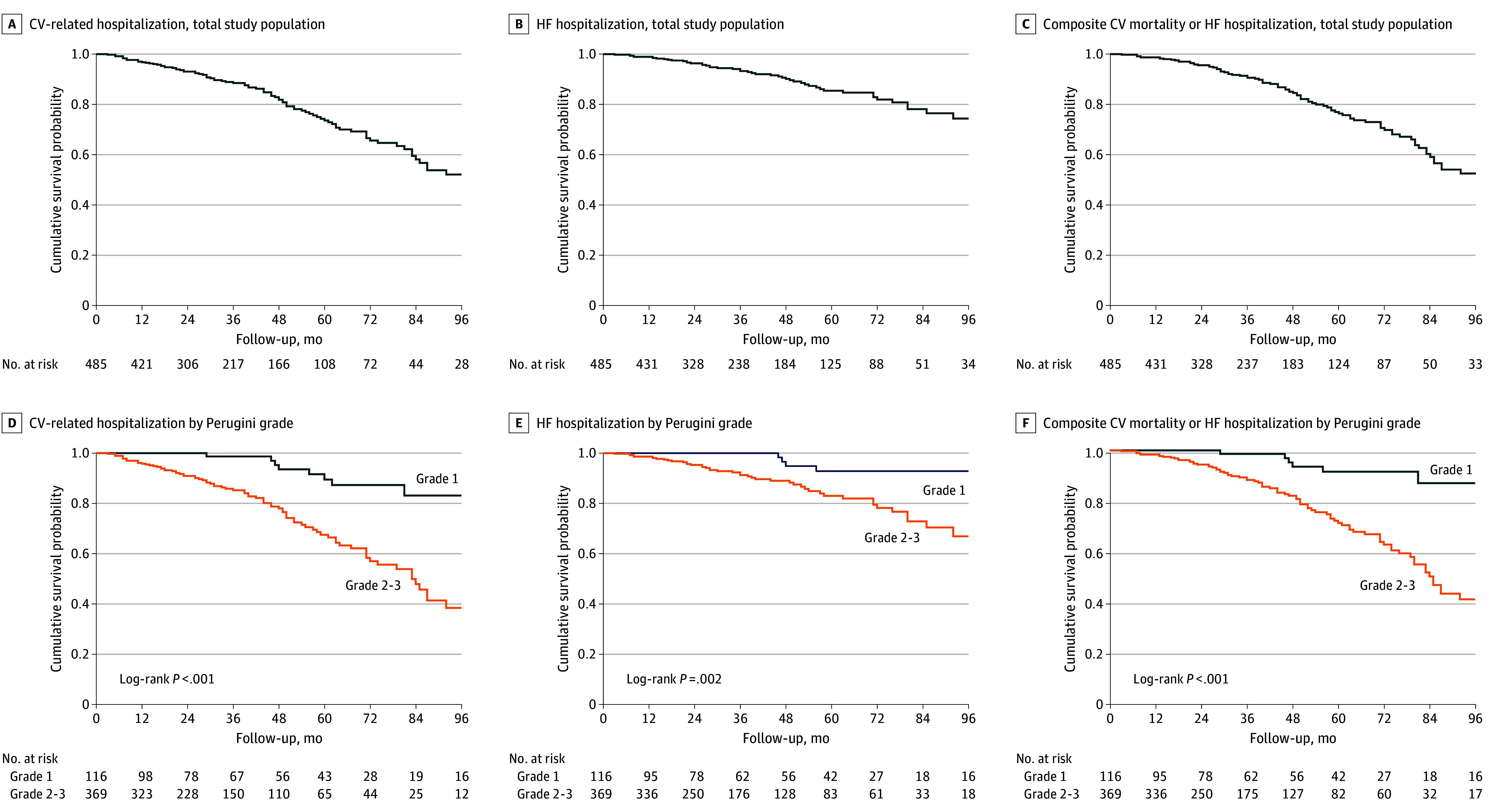
Unplanned Hospitalization for the Total Study Population and According to Grade of Cardiac Uptake at Diagnosis CV indicates cardiovascular; HF, heart failure.

Over a median follow-up period of 37 months (IQR, 20-64 months), there were 109 all-cause deaths (22.5%), 64 CV deaths (13.2%), and 93 composite events of CV death or HF hospitalization (19.2%) in the overall study population. There were no statistically significant differences in the all-cause mortality rate between patients with grade 1 vs grade 2 or 3 cardiac uptake (unadjusted HR, 1.25; 95% CI, 0.79-1.98; *P* = .33) (Figure 1 and Table 2). However, compared with patients with grade 1 uptake, patients with grade 2 or 3 uptake exhibited a significantly greater risk of CV mortality (unadjusted HR, 5.30; 95% CI, 1.92-14.65; *P* < .001) (Figure 1) and the composite of CV mortality or HF hospitalization (unadjusted HR, 5.80; 95% CI, 2.10-16.03; *P* < .001) (Figure 2) and a significantly lower risk of non-CV mortality (unadjusted HR, 0.46; 95% CI, 0.25-0.85; *P* = .01) (Figure 1). These findings were further confirmed with a sensitivity analysis in a cohort of 660 patients with asymptomatic ATTR cardiac amyloid infiltration phenotype treated with disease-modifying drugs or entered in a clinical trial during follow-up (eTables 1 and 2, eFigure 1 in Supplement 1).

**Table 2.** Event Rates of the Primary and Secondary Outcomes of the Study

Variable	Overall population (N = 485)			Perugini grade 1 (n = 116)			Perugini grades 2 and 3 (n = 369)			*P* value^a^
	Events	Events/100 patient-year	3-y Rate, % (95% CI)	Events	Events/100 patient-year	3-y Rate, % (95% CI)	Events	Events/100 patient-year	3-y Rate, % (95% CI)	
All-cause mortality	109	5.9 (5.9-7.1)	9.2 (6.6-12.7)	24	5.1 (3.4-7.6)	8.5 (4.1-17.0)	85	6.2 (5.0-7.7)	9.5 (6.5-13.7)	.11
CV mortality	64	3.5 (2.7-4.4)	4.2 (2.5-7.1)	5	1.0 (0.4-2.5)	1.4 (0.2-9.5)	59	4.3 (3.3-5.6)	5.1 (3.0-8.7)	<.001
HF hospitalization	49	2.8 (2.1-3.7)	6.7 (4.5-10.0)	4	0.8 (0.3-2.3)	0	45	3.5 (2.6-4.7)	8.7 (5.9-12.9)	.001
CV mortality and HF hospitalization	93	5.3 (4.3-6.5)	9.4 (6.7-13.0)	7	1.5 (0.7-3.0)	1.4 (0.2-9.5)	86	6.8 (5.5-8.3)	11.8 (8.4-16.4)	<.001
CV-related hospitalization	90	5.6 (4.5-6.9)	16.4 (12.9-20.6)	9	1.9 (1.0-3.7)	4.3 (1.6-11.3)	81	7.1 (5.7-8.8)	20.0 (15.7-25.3)	<.001
Non-CV mortality	45	2.4 (1.8-3.3)	5.1 (3.3-8.0)	19	4.0 (2.5-6.3)	7.2 (3.3-15.3)	26	2.0 (1.3-2.8)	4.6 (2.6-7.9)	.02
Outpatient development of HF	177	18.0 (15.6-20.9)	46.5 (40.8-52.5)	22	7.4 (4.9-11.3)	23.1 (14.8-35.1)	155	22.6 (19.4-26.5)	54.3 (47.7-61.3)	<.001
ODI	189	19.4 (16.8-22.4)	47.6 (42.0-53.5)	27	9.3 (6.4-13.6)	24.2 (15.9-35.7)	162	23.7 (20.3-27.6)	55.3 (48.8-60.0)	<.001
NT-proBNP progression	134	16.6 (14.0-19.7)	38.4 (32.4-45.0)	18	7.1 (4.5-11.2)	21.0 (12.8-33.4)	116	21.0 (17.6-25.2)	44.8 (37.5-52.8)	<.001
ODI and NT-proBNP progression	100	11.5 (9.5-14.0)	28.8 (23.2-35.3)	11	4.1 (2.3-7.4)	12.4 (6.3-23.7)	89	14.8 (12.0-18.2)	35.0 (28.0-43.2)	<.001

Abbreviations: CV, cardiovascular; HF, heart failure; NT-proBNP, N-terminal pro-B-type natriuretic peptide; ODI, outpatient diuretic initiation.

^a^ *P* value is measured using the log-rank test.

### Disease Progression in Asymptomatic Patients With ATTR Cardiac Amyloid Infiltration

Over the follow-up period, disease progression was assessed using multiple criteria, namely development of HF in an outpatient setting, ODI, NT-proBNP progression, a composite of ODI and NT-proBNP progression, unplanned CV-related hospitalization, and unplanned HF hospitalization. Event rates for all outcomes, measured as cumulative incidence either considering the total follow-up duration or at 3 years following diagnosis, favored patients with grade 1 vs grade 2 or 3 cardiac uptake (Table 2). At 3 years, compared with grade 1 uptake, patients with grade 2 or 3 uptake had greater development of HF (54.3% [95% CI, 47.7%-61.3%] vs 23.1% [95% CI, 14.8%-35.1%]), greater outpatient diuretic initiation and NT-proBNP progression (35.0% [95% CI, 28.0%-43.2%] vs 12.4% [95% CI, 6.3%-23.7%]), and greater HF hospitalization (8.7% [95% CI, 5.9%-12.9%] vs 0%) and unplanned CV hospitalization (20.0% [95% CI, 15.7%-25.3%] vs 4.3% [95% CI, 1.6%-11.3%]). These findings were further confirmed with a sensitivity analysis in a cohort of 660 patients with asymptomatic ATTR cardiac amyloid infiltration phenotype treated with disease-modifying drugs or entered in a clinical trial during follow-up (eTables 1 and 2, eFigure 2 in Supplement 1)

## Discussion

This cohort study represents the largest cohort of patients with asymptomatic ATTR cardiac amyloid infiltration to our knowledge. We found that (1) patients with grade 2 or 3 myocardial uptake exhibited significant cardiac functional and structural abnormalities, whereas patients with grade 1 uptake generally showed less abnormal cardiac structure and function; (2) the rate of development of symptomatic HF, diuretic initiation, NT-proBNP progression, HF hospitalization, and unplanned CV hospitalization was consistently higher in patients with grade 2 or 3 uptake compared with patients with grade 1 uptake; and (3) patients with grade 2 or 3 uptake had a 5-fold higher risk of CV death compared with patients with grade 1 uptake, whereas non-CV death was predominant among those with grade 1 uptake.

ATTR cardiac amyloid infiltration encompasses a wide spectrum of disease severity, ranging from amyloid deposits with no consequent cardiac structural and functional changes to established cardiomyopathy with the typical HF symptoms, elevated cardiac biomarkers, and echocardiographic evidence of cardiac dysfunction and morphologic changes. Bone scintigraphy is currently a key imaging modality in the noninvasive diagnostic algorithm for ATTR cardiac amyloidosis.^10,16^ Beyond its established diagnostic value,^10,16^ the degree of myocardial uptake of bone tracers has also been shown to provide a gross estimate of the presence and extent of cardiac amyloid infiltration.^17,18,19,20^

Our data show different disease stages within the spectrum of asymptomatic ATTR cardiac amyloid infiltration. Asymptomatic patients with grade 1 uptake showed less abnormal cardiac structure and function in almost all cases by echocardiogram with normal or only mildly elevated serum NT-proBNP concentrations. In contrast, patients with grade 2 or 3 uptake had structural and functional features on echocardiography typical of amyloid infiltration, namely increased left ventricular (LV) wall thickness and mass, reduced LV cavity size, atrial dilatation, increased LV filling pressures, preserved or mildly reduced LV ejection fraction, and reduced global longitudinal strain. These changes were also associated with significantly elevated serum NT-proBNP concentrations and a higher rate of atrial fibrillation (Table 1). Patients with asymptomatic ATTR cardiac amyloid infiltration at 3 years since diagnosis had significant morbidity and mortality. Patients with grade 2 or 3 cardiac uptake exhibited greater rates of disease progression across all metrics used compared with patients with grade 1 uptake. All-cause mortality rates were similar between patients with grade 2 or 3 uptake vs those with grade 1 uptake. However, patients with grade 2 or 3 uptake had a 5-fold higher risk of CV death compared with those with grade 1 uptake, in whom non-CV death was predominant.

These results have important implications for clinical practice and clinical trials. For clinical practice, the low rate of CV events and predominance of non-CV death in patients with grade 1 cardiac uptake, which is not likely to be modifiable by disease-modifying treatment, would support not treating asymptomatic patients with grade 1 uptake. However, patients with grade 2 or 3 cardiac uptake in our study, despite the absence of HF symptoms, exhibited structural and functional cardiac abnormalities with deranged cardiac biomarkers and an increased risk of disease progression, HF hospitalization, and CV mortality. Current European^21^ and US^22^ guidelines recommend treatment with tafamidis in patients with ATTR-CM and HF to reduce symptoms, CV hospitalizations, and mortality. Our findings call into question the current recommendations about whether asymptomatic patients with grade 2 or 3 myocardial uptake should also be treated with disease-modifying drugs. Notably, the US Food and Drug Administration commercial labeling for tafamidis only stipulates that a confirmed diagnosis of ATTR-CM be made without specifying further details with regard to HF symptomology. For clinical trials, the predominance of non-CV deaths in patients with grade 1 uptake is in keeping with the hypothesis that the risk of death in this group of patients is likely to be less modifiable. This hypothesis, coupled with our finding of a significantly lower rate of CV events, may imply that a clinical trial that includes these patients would require large numbers and long-term follow-up.

### Limitations

Our data need to be interpreted within the limitations of the study. This study was conducted in referral centers for the diagnosis and management of ATTR amyloidosis; therefore, referral and survival bias cannot be excluded. Even though exercise testing was not mandatory to confirm the absence of symptoms, the cohort’s low median NT-proBNP concentration and lack of diuretic therapy corroborate the exceedingly early disease stage in the context of the cohort’s advanced age and a significant proportion with atrial fibrillation and ischemic heart disease at diagnosis. Data on serum troponin were not included in the analysis due to substantial variability in the type of troponin (T or I) and the different laboratory assays used across participating centers.

Patients with grade 1 cardiac uptake pose diagnostic challenges in clinical practice. To reduce the risk of false-positive scintigraphy results, only patients with single-photon emission tomography/computed tomography were included. Furthermore, to reduce the risk of including patients with non-ATTR amyloidosis, particularly light-chain amyloidosis (the other most common cause of grade 1 cardiac uptake), we limited inclusion to patients without PCD or required histologic confirmation of ATTR amyloid in those with PCD. Decisions concerning initiation of diuretic treatment for HF onset were made following each clinical assessment on a case-by-case basis by the managing clinician.

Deaths were not adjudicated blindly. There was evidence of an increase in non-CV mortality in patients with grade 1 cardiac uptake, which may be associated with multiple factors, including the bimodal distribution of age at diagnosis among patients with grade 1 uptake (with a higher proportion of older patients) (eFigure 3 in Supplement 1) and the referral pattern of patients with grade 1 uptake, where a higher proportion of patients had bone scintigraphy as part of diagnostic workup for cancer (more than one-quarter of cases). The absolute number of CV deaths and HF hospitalizations was low in patients with grade 1 uptake, with none experiencing HF hospitalization within 3 years of diagnosis. While these findings reflect the generally favorable natural history of patients with grade 1 uptake, the small number of events warrants cautious interpretation of results.

Cardiac scintigraphy was performed with different bone tracers, although all were validated for diagnostic purposes according to center local protocols. Serial data on cardiac scintigraphy with bone tracers were not available. Cardiac magnetic resonance imaging data also were not available for this analysis.

## Conclusions

Asymptomatic ATTR cardiac amyloid infiltration is being increasingly recognized in clinical practice. Patients with grade 2 or 3 cardiac uptake in this cohort study had substantial cardiac functional and structural abnormalities, consistent with ATTR-CM, whereas patients with grade 1 uptake mostly had less abnormal cardiac structure and function, indicating initial ATTR cardiac amyloid infiltration without overt cardiomyopathy. Patients with grade 2 or 3 uptake had significant rates of disease progression and greater risks of CV mortality, HF hospitalization, and CV-related hospitalization compared with patients with grade 1 uptake. In the absence of randomized clinical trials, these data support the use of disease-modifying treatments in asymptomatic patients with grade 2 or 3 cardiac uptake and highlight the need of large-scale future studies to assess the clinical importance of grade 1 uptake and the role of disease-modifying treatment in these patients.

## Notes

Supplement 1.
eResults
**eTable 1.** Baseline Characteristics of the Full Study Population, Including Patients Treated With Disease-Modifying Therapy and Enrolled in Clinical Trials According to Perugini Grade **eTable 2.** Event Rates for Primary and Secondary Outcomes of the Untreated Study Population Not Enrolled in Clinical Trials **eFigure 1.** Kaplan-Meier Curves for All-Cause Mortality, CV Mortality and Non- CV Mortality According to Grade of Cardiac Uptake at Diagnosis **eFigure 2.** Kaplan-Meier Curves for Unplanned CV-Related Hospitalization, HF Hospitalization and the Composite of CV Mortality and HF Hospitalization According to Grade of Cardiac Uptake at Diagnosis **eFigure 3.** Distribution of Age at Diagnosis of Asymptomatic ATTR Cardiac Amyloid Infiltration in the Study Population According to Perugini Grade

Supplement 2.
Data Sharing Statement
