# Supplementary material for: Clinical Phenotype and Prognosis of Asymptomatic Patients With Transthyretin Cardiac Amyloid Infiltration
Source: JAMA Cardiol. 2025 Jan 22;10(5):437–45. doi: 10.1001/jamacardio.2024.5221 (PMC12079285; doi:10.1001/jamacardio.2024.5221)
Supplement: Supplement 1. — eResults eTable 1. Baseline Characteristics of the Full Study Population, Including Patients Treated With Disease-Modifying Therapy and Enrolled in Clinical Trials According to Perugini Grade eTable 2. Event Rates for Primary and Secondary Outcomes of the Untreated Study Population Not Enrolled in Clinical Trials eFigure 1. Kaplan-Meier Curves for All-Cause Mortality, CV Mortality and Non- CV Mortality According to Grade of Cardiac Uptake at Diagnosis eFigure 2. Kaplan-Meier Curves for Unplanned CV-Related Hospitalization, HF Hospitalization and the Composite of CV Mortality and HF Hospitalization According to Grade of Cardiac Uptake at Diagnosis eFigure 3. Distribution of Age at Diagnosis of Asymptomatic ATTR Cardiac Amyloid Infiltration in the Study Population According to Perugini Grade [file jamacardiol-e245221-s001.pdf]

Supplemental Online Content

Porcari A, Razvi Y, Cappelli F, et al. Clinical phenotype and prognosis of asymptomatic patients with transthyretin cardiac amyloid infiltration. *JAMA Cardiol*. Published online January 22, 2025. doi:10.1001/jamacardio.2024.5221

eResults

eTable 1. Baseline Characteristics of the Full Study Population, Including Patients Treated With Disease-Modifying Therapy and Enrolled in Clinical Trials According to Perugini Grade

eTable 2. Event Rates for Primary and Secondary Outcomes of the Untreated Study Population Not Enrolled in Clinical Trials

eFigure 1. Kaplan-Meier Curves for All-Cause Mortality, CV Mortality and Non- CV Mortality According to Grade of Cardiac Uptake at Diagnosis

eFigure 2. Kaplan-Meier Curves for Unplanned CV-Related Hospitalization, HF Hospitalization and the Composite of CV Mortality and HF Hospitalization According to Grade of Cardiac Uptake at Diagnosis

eFigure 3. Distribution of Age at Diagnosis of Asymptomatic ATTR Cardiac Amyloid Infiltration in the Study Population According to Perugini Grade

This supplemental material has been provided by the authors to give readers additional information about their work.

© 2025 American Medical Association. All rights reserved, including those for text and data mining, AI training, and similar technologies.

eResults

The population comprised 142 patients with ATTRv amyloidosis: 26 with p.V142I-associated ATTR amyloidosis, 36 with p.T80A-associated ATTR amyloidosis, and 80 patients with non-p.V142I non-p.T80A-associated ATTR amyloidosis (p.Ile88Leu, 20 patients; p.Val50Met, 18 patients; p.Gly67Val, 4 patients; p.His129Arg, 3 patients, two patients each with p.Ala117Ser, p.Ala56Pro, p.Ala69Thr, Glu109Gln, p.His110Asp, p.Ile127Phe, p.Ile127Val, p.Phe84Ile, p.Tyr134Cys, p.Ile104Ser; and one patient each with, p.Ala98Ser, p.Arg54Thr, p.Arg25His, p.Glu65Gln, p.Glu129Gln, p.Glu74Gly, p.Glu94Gln, p.Glu102Lys, p.Gly87Glu, p.Gln723Lys, p.Ala129Ser, p.Tyr98Phe, p.Val40Ile, p.Val123Leu, p.Val124Leu, p.Tyr126Ser, p.Tyr89Phe).

© 2025 American Medical Association. All rights reserved, including those for text and data mining, AI training, and similar technologies.

eTable 1. Baseline Characteristics of the Full Study Population, Including Patients Treated With Disease-Modifying Therapy and Enrolled in Clinical Trials According to Perugini Grade

Parameters	Missing	Grade 1 (n=122)	Grade 2-3 (n=538)	p value
Age, years	0	74.2±11.7	74.1±9.3	0.303
Sex (male)	0	99 (80.5%)	475 (88.5%)	**0.018**
SBP, mmHg	11	132 [123-149]	130 [120-146]	0.062
ATTRv amyloidosis	0	37 (30.1%)	105 (19.6%)	**0.010**
Atrial fibrillation	0	24 (19.5%)	177 (33%)	**0.003**
IHD	0	23 (18.7%)	89 (16.6%)	0.57
Diabetes mellitus	0	17 (13.8%)	60 (11.2%)	0.41
Hypertension	0	48 (39%)	264 (49.2%)	**0.042**
Previous stroke/TIA	0	7 (5.7%)	44 (8.2%)	0.35
**Heart failure severity**				
NYHA class	0			-
1		122 (100%)	538 (100%)	
2		0 (0%)	0 (0%)	
3		0 (0%)	0 (0%)	
4		0 (0%)	0 (0%)	
NAC stage	0			**0.001**
1a		81 (66.4%)	263 (49%)	
1b		37 (30.1%)	249 (46.4%)	
2		4 (3.3%)	25 (4.7%)	
3		1 (0.8%)*	0 (0%)	
NT-proBNP, ng/L	0	279 [100-882]	677 [349-1152]	**<0.001**
eGFR, ml/min/1.73m²	0	74 [63-89]	74 [59-91]	0.18
**Echocardiographic parameters**				
IVS, mm	0	12.6±2.8	16.1±3.0	**<0.001**
PW, mm	0	11.6±2.5	14.7±2.7	**<0.001**
RWT	98	0.44 [0.41-0.52]	0.72 [0.57-0.85]	**<0.001**
LVEF, %	0	58.7±8.6	56.9±8.6	**0.017**
LVEF ≤50%	0	12 (9.8%)	95 (17.7%)	**0.031**
LVEF ≤40%	0	4 (3.3%)	24 (4.5%)	0.54
LV-GLS,%	64	-19.0 [-16.0 to -20.2]	-14.8 [-11.8 to -17.9]	**<0.001**
E/e’	53	9.8±3.9	13.4±5.1	**<0.001**
LA area, cm2	77	19.3±4.5	26.5±9.0	**<0.001**
RA area, cm2	81	17.0±4.2	20.8±6.8	**<0.001**
TAPSE, mm	77	21.0±2.9	20.0±4.2	**0.008**
**Medications**				
Loop diuretic	0	0 (0%)	0 (0%)	-
Beta-blockers	0	19 (15.4%)	175 (32.6%)	**<0.001**
ACEi/ARB/ARNIs	0	26 (21.3%)	233 (43.5%)	**<0.001**
MRAs	0	0 (0%)	33 (6.1%)	**0.005**
SGLT2-i	0	3 (2.4%)	9 (1.7%)	0.56

Data are presented as frequencies (%), mean +/- standard deviation and median [interquartile range]. **Legend**:, eGFR, estimated Glomerular Filtration Rate; hATTR, hereditary Transthyretin Amyloidosis; IHD, Ischemic Heart Disease; LVEF, Left Ventricle Ejection Fraction; MRAs, Mineralocorticoid Receptor Antagonists; MWT, Maximal Wall Thickness; NAC, National Amyloidosis Centre; NT-proBNP, N-terminal pro-Brain Natriuretic Peptide; NYHA, New York Heart Association;

© 2025 American Medical Association. All rights reserved, including those for text and data mining, AI training, and similar technologies.

PWT, Posterior Wall Thickness; SBP, Systolic Blood Pressure; SD, mean Standardised Difference; SGLT2, Sodium-glucose Cotrasport-2; TAPSE, Tricuspid Annular Plane Systolic Excursion; wtATTR, wild-type Transthyretin Amyloidosis. * a single patient on haemodialysis for end stage renal disease.

© 2025 American Medical Association. All rights reserved, including those for text and data mining, AI training, and similar technologies.

eTable 2. Event Rates for Primary and Secondary Outcomes of the Untreated Study Population Not Enrolled in Clinical Trials

Variables	Overall population (n=660)			Grade 1 (n=122)			Grade 2 and 3 (n=538)			p value
	events	events/100 patient-yr	3-yr rate (95% CI)	events	events/100 patient-yr	3-yr rate (95% CI)	events	events/100 patient-yr	3-yr rate (95% CI)	
All-cause mortality	115	4.6 (3.8-5.6)	7.4% (5.3-10.1)	26	5.0 (3.4–7.3)	7.7% (3.7-15.5)	89	4.5 (3.7–5.6)	7.3% (5.1-10.5)	0.89
CV mortality	64	2.6 (2.0-3.3)	3.4% (2-5.6)	5	1.0 (0.4–2.3)	1.3% (0.2-8.5)	59	3.0 (2.3–3.9)	3.9% (2.3-6.5)	**0.003**
HF hospitalization	59	2.5 (1.9-3.2)	5.5% (3.7-8)	4	0.8 (0.3-2.0)	0%	55	3.0 (2.2-3.8)	6.6% (4.5-9.6)	**0.005**
CV mortality or HF hospitalization	105	4.4 (3.7-5.4)	8% (5.9-11)	7	1.4 (0.6–2.9)	1.3% (0.2-8.8)	98	5.3 (4.3–6.4)	9.5% (7-13)	**0.001**
CV-related hospitalization	115	5.3 (4.4-6.4)	14% (11.6-17.7)	10	2.0 (1.1-3.7)	4% (1.5-10.5)	105	6.3 (5.2-7.6)	16.7% (13-21)	**<0.001**
Non-CV mortality	51	2.0 (1.6-2.7)	4.1% (2.7-6.3)	21	4.0 (2.6 –6.2)	6.5% (3-14)	30	1.5 (1.0–2.2)	3.6% (2.1-6)	**0.001**
Outpatient development of HF	237	17.2 (15.1-19.5)	45% (40-50)	23	6.9 (4.6-10.4)	23% (15-35)	214	20.5 (17.9-23.4)	50% (44-56)	**<0.001**
ODI	263	19.9 (17.6-22.4)	49% (44-54)	28	9.2 (6.4-13.4)	25% (16-36)	235	23.0 (20.3-26.2)	54% (49-60)	**<0.001**
NT-proBNP progression	180	15.3 (13.2-17.8)	37% (32-43)	18	6.7 (4.2-10.7)	21% (13-34)	162	17.9 (15.3-20.9)	41% (36-48)	**<0.001**
ODI and NT-proBNP progression	131	10.8 (9.1-12.8)	28% (23-34)	11	3.9 (2.1-7.0)	12.6% (6.5-24)	120	12.9 (10.8-15.4)	32.5% (27-39)	**<0.001**

Legend: CI, Confidence Interval; CV, Cardiovascular; HF, Heart Failure; HR, Hazard Ratio; n, number; ODI, Outpatient Diuretic Inititation; yr, year. *p-value is measured using the log-rank test.

© 2025 American Medical Association. All rights reserved, including those for text and data mining, AI training, and similar technologies.

eFigure 1. Kaplan-Meier Curves for All-Cause Mortality, CV Mortality and Non- CV Mortality According to Grade of Cardiac Uptake at Diagnosis

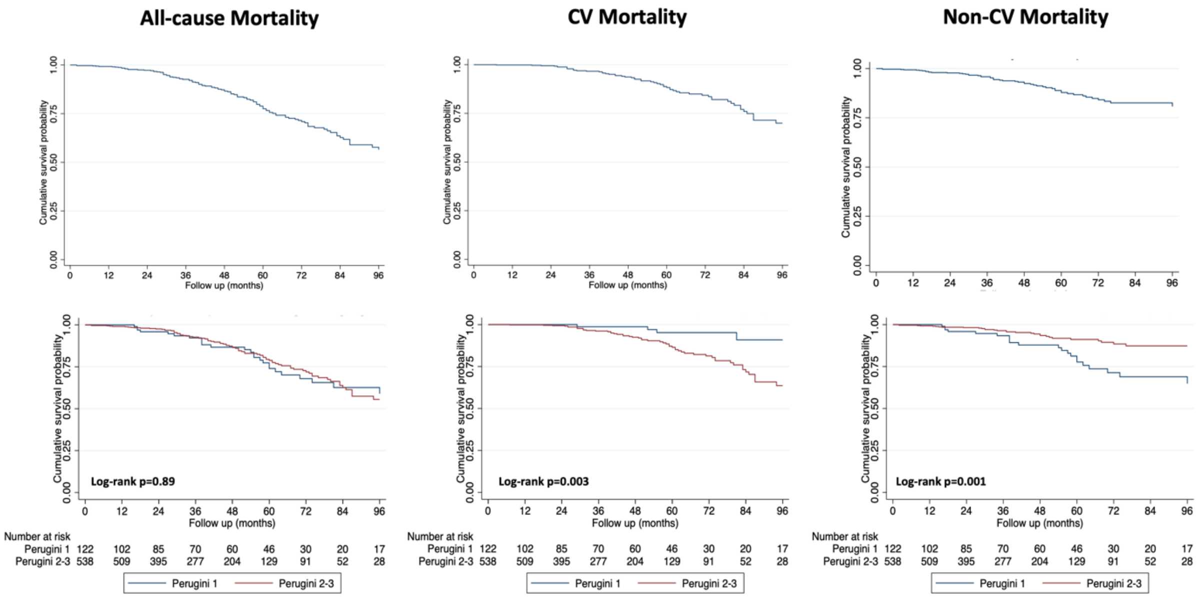

Top row shows cumulative survival probability in the total study population. **Legend:** CV, Cardiovascular.

© 2025 American Medical Association. All rights reserved, including those for text and data mining, AI training, and similar technologies.

eFigure 2. Kaplan-Meier Curves for Unplanned CV-Related Hospitalization, HF Hospitalization and the Composite of CV Mortality and HF Hospitalization According to Grade of Cardiac Uptake at Diagnosis

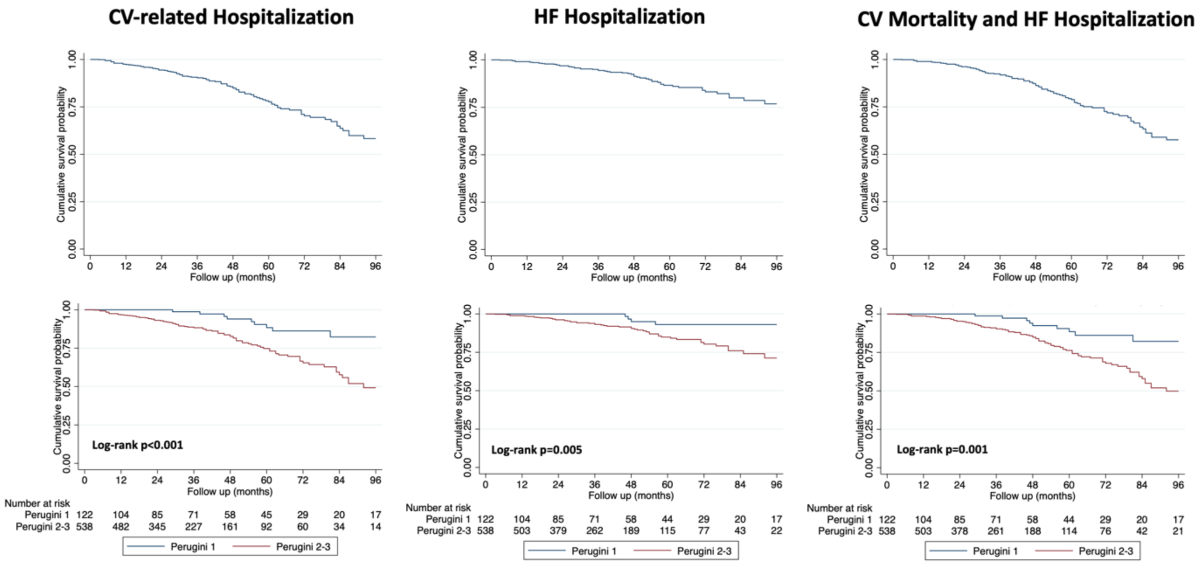

Top row shows cumulative survival probability in the total study population. **Legend:** CV, Cardiovascular; HF, Heart Failure.

© 2025 American Medical Association. All rights reserved, including those for text and data mining, AI training, and similar technologies.

eFigure 3. Distribution of Age at Diagnosis of Asymptomatic ATTR Cardiac Amyloid Infiltration in the Study Population According to Perugini Grade

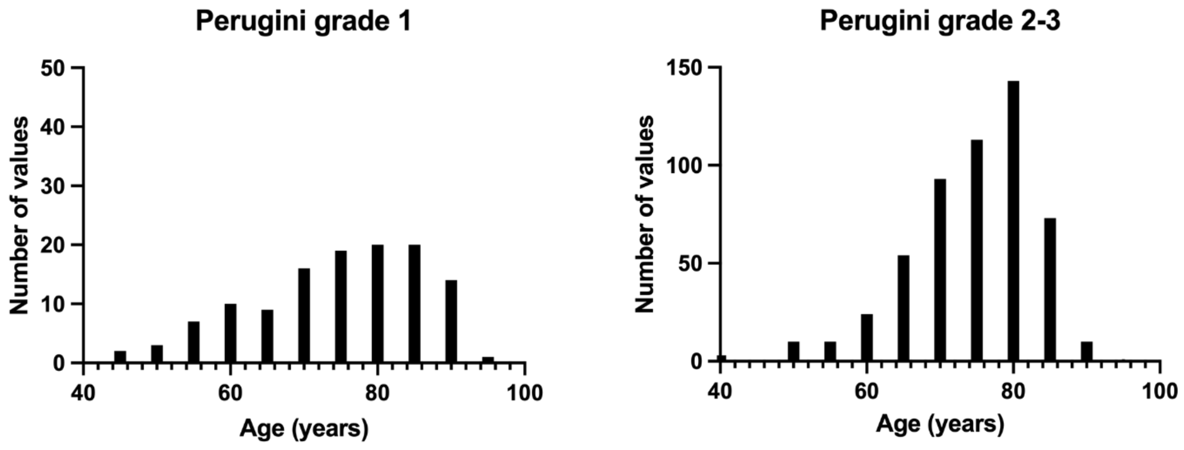

© 2025 American Medical Association. All rights reserved, including those for text and data mining, AI training, and similar technologies.
