# Supplementary material for: Clinical Phenotype and Prognosis of Asymptomatic Patients With Transthyretin Cardiac Amyloid Infiltration
Source: JAMA Cardiol. 2025 Jan 22;10(5):437–45. doi: 10.1001/jamacardio.2024.5221 (PMC12079285; doi:10.1001/jamacardio.2024.5221)
Supplement: Supplement 2. — Data Sharing Statement [file jamacardiol-e245221-s002.pdf]

Data Sharing Statement

Porcari. Clinical Phenotype and Prognosis of Asymptomatic Patients With Transthyretin Cardiac Amyloid Infiltration. *JAMA Cardiol*. Published January 22, 2025.
doi:10.1001/jamacardio.2024.5221

Data
Data available: No

Additional Information
Explanation for why data not available: The data underlying this article cannot be shared publicly because of the privacy of individuals who participated in the study. The data will be shared on reasonable request to the corresponding author.
